# X-ray Fluorescence Nanotomography of Single Bacteria with a Sub-15 nm Beam

**DOI:** 10.1038/s41598-018-31461-y

**Published:** 2018-09-07

**Authors:** Tiffany W. Victor, Lindsey M. Easthon, Mingyuan Ge, Katherine H. O’Toole, Randy J. Smith, Xiaojing Huang, Hanfei Yan, Karen N. Allen, Yong S. Chu, Lisa M. Miller

**Affiliations:** 1 Department of Chemistry, Stony Brook University, Stony Brook, NY 11794 USA; 2 Department of Chemistry, Boston University, Boston, MA 02215 USA; 3 National Synchrotron Light Source II, Brookhaven National Laboratory, Upton, NY 11973 USA

## Abstract

X-ray Fluorescence (XRF) microscopy is a growing approach for imaging the trace element concentration, distribution, and speciation in biological cells at the nanoscale. Moreover, three-dimensional nanotomography provides the added advantage of imaging subcellular structure and chemical identity in three dimensions without the need for staining or sectioning of cells. To date, technical challenges in X-ray optics, sample preparation, and detection sensitivity have limited the use of XRF nanotomography in this area. Here, XRF nanotomography was used to image the elemental distribution in individual *E*. *coli* bacterial cells using a sub-15 nm beam at the Hard X-ray Nanoprobe beamline (HXN, 3-ID) at NSLS-II. These measurements were simultaneously combined with ptychography to image structural components of the cells. The cells were embedded in small (3–20 µm) sodium chloride crystals, which provided a non-aqueous matrix to retain the three-dimensional structure of the *E*. *coli* while collecting data at room temperature. Results showed a generally uniform distribution of calcium in the cells, but an inhomogeneous zinc distribution, most notably with concentrated regions of zinc at the polar ends of the cells. This work demonstrates that simultaneous two-dimensional ptychography and XRF nanotomography can be performed with a sub-15 nm beam size on unfrozen biological cells to co-localize elemental distribution and nanostructure simultaneously.

## Introduction

Trace elements are essential for carrying out biochemical reactions and act as structural components in cells. The appreciation of the important roles that trace elements, especially metals, play in cell metabolism has resulted in an increase in the number of studies in this area^1–5^. However, to truly understand the functions of trace elements in cells and tissues, the distribution within a cell must be imaged and quantified in the native cellular environment, which has proven to be difficult.

Hard XRF microscopy is a well-suited microanalytical technique for assessing the elemental distribution in a wide range of materials including whole cells and tissues^6–8^. In XRF microscopy, multiple elements can be mapped simultaneously, which provides precise elemental co-localization and speciation. High detection sensitivity down to sub-parts-per-million has been demonstrated in biological studies^9,10^.

To understand the functions of trace elements in biological cells, the X-ray probe must resolve subcellular compartments on the sub-micron to nanometer scale such as mammalian nuclei, mitochondria, and cell membranes, which measure ~6 μm, ~1 μm and ~10 nm, respectively^11^. In prokaryotic cells such as *E*. *coli* (1 μm × 3 μm), the outer and inner cell membranes are each ~10–15 nm wide, while ribosomes are about 20 nm in size^12^.

Nanoscale imaging with X-rays requires high-resolution nano-focusing optics like Kirkpatrick-Baez mirrors^13–15^ and Fresnel zone plate optics^16^. A more sophisticated, higher-resolving, nano-focusing approach is to use Multilayer Laue Lenses (MLLs)^17–19^, incorporated into a specialized X-ray microscope located at a beamline with infrastructure for good vibration isolation and thermal stability^20–22^.

It is well-accepted that X-ray imaging of living biological specimens can be a challenge due to radiation-induced damage, so cells are frequently imaged in the fixed/dried or frozen-hydrated state^23^. While fixed/dried cells are least subject to radiation damage from water hydrolysis and free radical production, the subcellular structure can be compromised, especially at the nanoscale. Frozen-hydrated cells represent the most natural state of the cell while reducing the effects of radiation damage; however, this method requires the cells to be flash frozen, e.g. with a plunge freezer, and imaged in a cryostage. Since optical configurations of nanotomography instruments with MLLs^21^ have very short working distances and rotation constraints, integration of a cryostage is a significant technical challenge. In addition, since the trace elements are typically micro- to nanomolar in concentration, sensitive detectors with a large solid angle situated close to the sample are required for quantification, further increasing technical complexity.

Whereas two-dimensional XRF microscopy is the most straightforward approach for imaging cells and tissues, these two-dimensional data can be ambiguous when imaging three-dimensional objects, e.g. when aiming to differentiate between elements located on the surface of a cell from those distributed throughout the cell. At present, only a few three-dimensional XRF tomography studies from whole biological specimens have been reported. Kim *et al*. have studied the role of the vacuolar iron uptake transporter in the uptake and distribution of iron in the *Arabidopsis* seed, which was performed at 12 μm spatial resolution^24^. More recently, de Jonge *et al*. performed three-dimensional XRF tomography of a whole diatom, *Cyclotella meneghiniana*, at 400 nm spatial resolution using 150 nm voxels over a 15 μm field of view^9^. This work elegantly showed the distribution of several species including silicon and zinc within the organelles including the siliceous frustule, a diatom’s cell wall. Most surprising was the detection of Fe and Mn rings in the cell wall that would not have been detectable using only two-dimensional projections.

To date, XRF nanotomography has been limited by sub-micron spatial resolution and weak absorption contrast in biological cells, inhibiting its ability to distinguish the ultra-structures of cell organelles^25^. In contrast, coherent diffraction imaging techniques, such as ptychography, can be used to image the fine structural components in samples^26^ but cannot determine the distribution of trace elements. However, by combining XRF nanotomography and ptychography, it is possible to obtain complementary data on elemental and structural composition, respectively, at the nanoscale from biological cells^8,27^. The results described here represent a combination of two-dimensional ptychography and XRF nanotomography performed on *E*. *coli* cells using a MLL microscope with a sub-15 nm focused beam.

## Methods

### Cell culture

LB media (10 mL) was inoculated with a single colony of BL21 (DE3) *E*. *coli* cells and the culture grown at 37 °C with shaking to an OD of 0.6–0.8. Cells were harvested in 1 mL aliquots via centrifugation at 3,500 RPM, flash frozen in liquid nitrogen, and stored at −80 °C.

### Sample preparation, light microscopy and scanning electron microscopy

**Figure 1:**
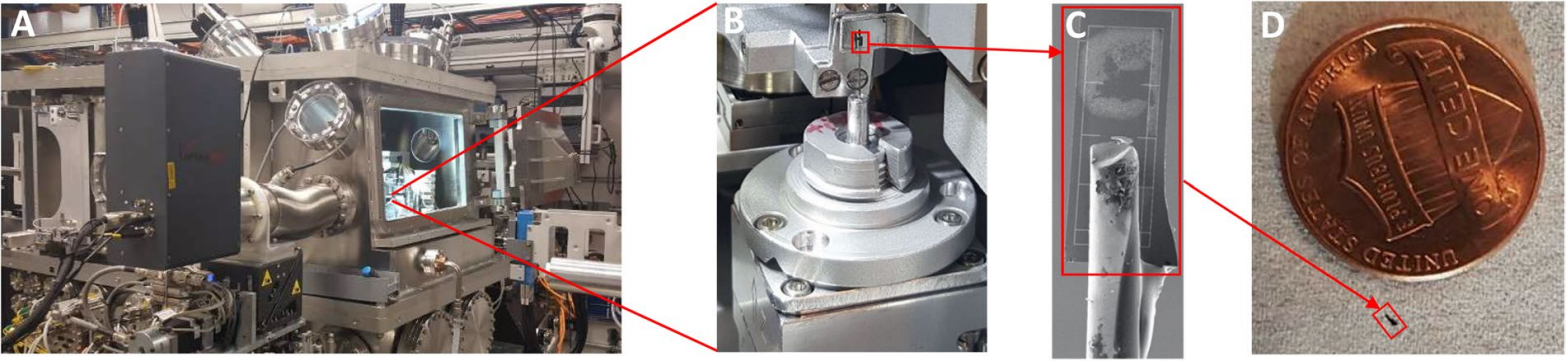
(**A**) X-ray microscope at the Hard X-ray Nanoprobe (HXN, 3-ID) beamline at NSLS-II. (**B**) Sample mounted on the rotation stage for tomography, (**C**) *E*. *coli* bacteria deposited on a Si substrate (“diving board”) that was mounted to an insect pin. (**D**) Size comparison between the Si substrate and a US penny. Substrate is 1.4 × 0.4 mm.

A 20 μl solution containing 2 μM NaCl, 0.01 OD *E*. *coli* cells, and 0.025 OD 100 nm gold nanoballs (CytoDiagnostics, #GRF-100-20) was mixed thoroughly. The gold nanoballs were added as fiducial markers for the tomography reconstruction. One microliter of this solution was pipetted onto a silicon chip sample holder with Cr fiduciary grids (Fig. 1C,D), also known as a “diving board” (substrate area 1.4 mm by 0.4 mm with a 10 μm thickness) (Norcada NCT4155P-III-Cr), and allowed to dry overnight. The diving board was then glued onto a stainless-steel insect pin (0.2 mm thick, 16.5 mm tall) (Fig. 1C) and mounted on the sample mount (Fig. 1B) for the X-ray microscope (Fig. 1A). The diving board has 5 μm wide chromium-patterned gridlines for sample alignment in the X-ray microscope. Images of cells prepared on the diving boards were taken using both a visible light microscope (Nikon Eclipse LVDIA-N) and a scanning electron microscope (SEM, JEOL 300) for cell identification, correlation, and navigation during data acquisition at the beamline.

### Synchrotron X-ray fluorescence microscopy and ptychography measurements

The distribution of elements was mapped using nano-Mii (Nanoscale Multimodal Imaging Instrument) at beamline 3-ID (HXN) at the National Synchrotron Light Source II (Fig. 1A). Beamline 3-ID provides a scanning X-ray microscope capable of multimodal imaging including absorption, fluorescence, differential phase contrast, and ptychography^28^. In this study, a 12 keV incident beam was focused to a sub-15 nm spot (14 nm horizontal and 12 nm vertical) using Multilayer Laue Lenses (MLLs). The coherent illumination was selected by a 50 × 30 µm secondary source aperture placed about 15 m in front of the nanofocusing optics. A three-element silicon drift detector (Vortex-ME3) was placed perpendicular to the incident beam to collect the fluorescence signals, and the transmitted scattering data for differential phase contrast or ptychography reconstructions were acquired by using a pixelated area detector (Merlin) with a pixel size of 55 µm at a distance of 0.5 m downstream the sample.

Two-dimensional fluorescence/ptychography scans, via continuous fly-scan, were performed using a step size of 20 nm/pixel and a dwell time of 250 ms. For the tomography data set, the cells were scanned from -39 to 132 degrees at 3 degree intervals using a dwell time of 100 ms and a 20 nm step size. Each projection was ~14 min. After each rotation interval, a short coarse scan (~1 min) was used to center the sample in the field of view of the projection image, resulting in a total imaging time for a complete cell of approximately 15 hours, excluding overhead.

The fitting of the X-ray fluorescence data was accomplished using PyXRF, an X-ray fluorescence analysis package developed at NSLS-II^29^. For the fitting process, the summed spectrum was first fitted using the non-linear least squares method to determine the global parameters such as the energy-calibration values, widths of the global peaks, and parameters related to the Compton and elastic peaks. Once the correct global parameters were obtained, the peak area of each element under the XRF spectrum of each single pixel was then fitted using a nonnegative least squares approach. TomoPy was used for the tomography reconstruction of the data sets^30^. The projection images were aligned by calculating the cross-correlation of a pair of images taken at successive projection angles. The aligned image stack was reconstructed using the maximum-likelihood method coded in the TomoPy package^30^, and 20 iterations were used to reconstruct each slice. To correct for any attenuation (due to the thickness of the diving board, since the XRF photons are detected through the diving board for some projections), the total intensity of the Zn fluorescence images was normalized to be constant for all the images taken at different projection angles.

For the ptychography reconstruction, a 96 × 96 array was cropped from the recorded raw dataset, which gave a reconstruction pixel size of about 10 nm. The cropped data array was fed into 100 iterations of a difference map algorithm for ptychography reconstruction^31^. From the preliminary reconstruction result, we noticed that the measurement was not performed exactly at the focal plane (due to run-out error of the rotary stage). In order to achieve the best reconstruction image quality, the exact illumination function at the measurement plane was searched by running the reconstruction process using a series of propagated wavefronts as the initial probe guesses, and the one that gave the best contrast was selected. A series of illumination functions propagated to various distances from the focus were tested, and the one that gave the best reconstruction image quality was selected. To assist the reconstruction convergence, the allowable ranges for the amplitude and phase parts of the *E*. *coli* image were constrained to [0.95, 1.0] and [−0.1, 0.0], respectively. The last 20 iterations were averaged to give the final reconstructed image.

## Results

**Figure 2:**
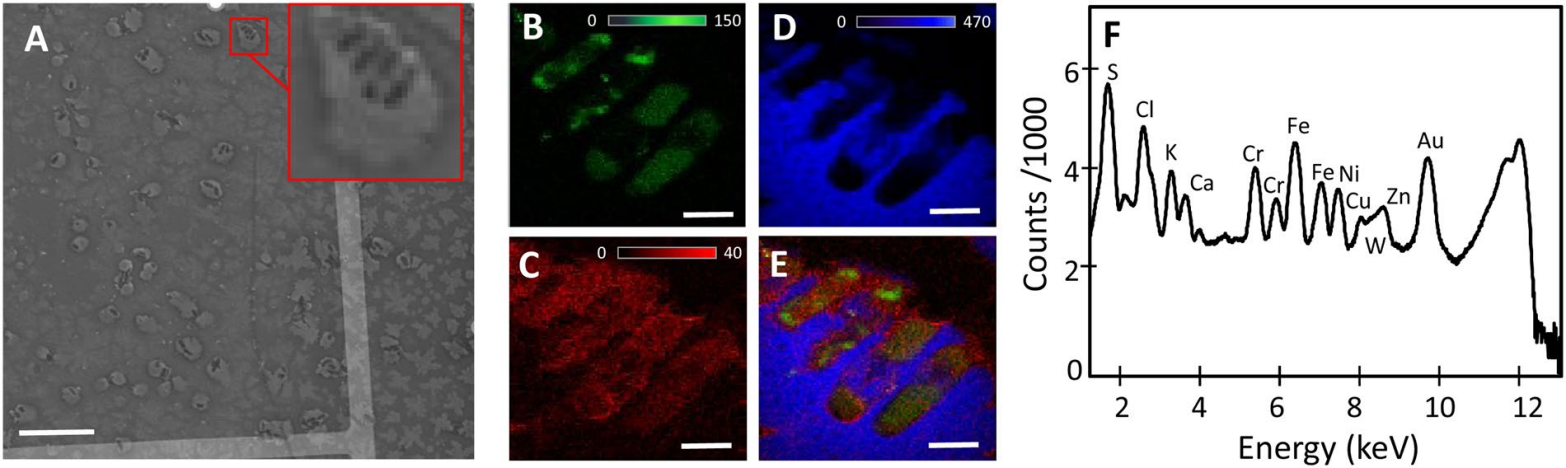
(**A**) SEM image showing *E*. *coli* bacteria embedded in NaCl crystals on the Si substrate with Cr-patterned fiducial grids. Cells appear as negative (dark) contrast regions in images. Scale bar is 20 μm. XRF images showing (**B**) Zn, (**C**) Ca, and (**D**) Cl distribution in the *E*. *coli* cells. (**E**) XRF image showing the co-localization of Zn, Ca and Cl in the *E*. *coli* cells. (**F**) Average XRF spectrum from the sample. Scale bar is 1 μm in (**B**–**E**).

*E*. *coli* cells were deposited on the diving board and examined under a visible light microscope in epi-brightfield mode followed by visualization using SEM. The SEM image (Fig. 2A**)** displays *E*. *coli* cells (typically 1 μm × 3 μm in size) embedded in small sodium chloride crystals (3–20 μm) that formed on the silicon substrate, where the cells appear as dark (negative contrast) regions in the crystals. The inset in Fig. 2A shows a magnified view of a group of cells lined up side-by-side within a sodium chloride crystal.

Two-dimensional elemental images (Fig. 2B–E**)** were taken using XRF microscopy, mapping the distribution and co-localization of zinc, calcium, and chlorine. The chlorine image (Fig. 2D) shows the location of the sodium chloride crystals and the dark regions indicate the areas occupied by the cells embedded in the crystal, which correlates well with the SEM image in Fig. 2A (inset). Most notably, zinc exhibits an inhomogeneous distribution (Fig. 2B) whereas a more uniform distribution of calcium (Fig. 2C) is observed throughout the cell. An XRF spectrum showing elements present in the sample is seen in Fig. 2F. It should be noted that peaks from Fe, Ni, and W are contributions from the microscope, the Cr signal arises from the bars on the substrate, and the Au signal comes from the nanoball fiducials.

**Figure 3:**
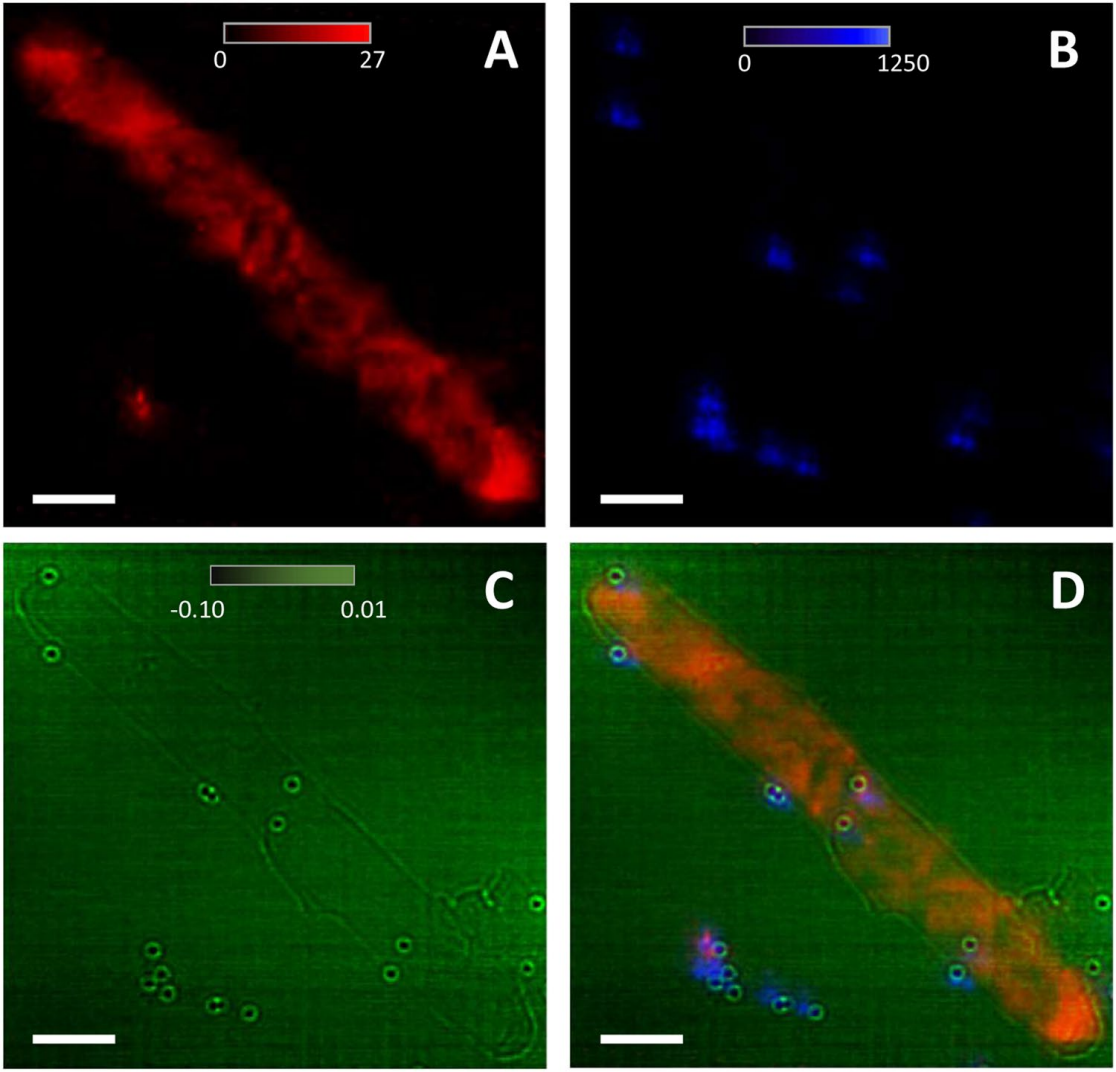
XRF images showing the (**A**) Zn and (**B**) Au distribution in several *E*. *coli* cells lined end-to-end. (**C**) Ptychography reconstructed phase image of the cells from (**A**) showing the cell boundaries and the 100 nm Au nanoballs that were added to the cell solution as fiducial markers for alignment and tomography reconstruction. (**D**) Overlay of the ptychography and XRF images from (**A**–**C**). Scale bar is 1 μm.

In Fig. 3A, the two-dimensional zinc distribution is shown in a series of two cells lined end-to-end. Again, the distribution of zinc is uneven within the cells and varies from cell to cell. The gold elemental map (Fig. 3B) was used to localize the gold nanoballs, which were added to the cell solution as fiducial markers for the alignment and reconstruction of the tomography data. These gold nanoballs are also visible in the ptychography reconstructed phase image (Fig. 3C) along with the clear outline of two cells that appear to be dividing. An overlay of the ptychography and XRF images can be seen in Fig. 3D. It should be noted that the edge enhanced contrast in the ptychography image is most likely due to the propagation uncertainty along the beam direction^32^. As the reconstruction plane in the ptychography method is determined by the illumination function^26^, the object transmission functions that propagate within the depth of focus range (±2 μm for MLLs) are allowed in the phase-retrieval process. For an object with extremely small contrast, which is the case for this study, the edge-enhanced artifact is more pronounced than the objects with higher contrast.

**Figure 4:**
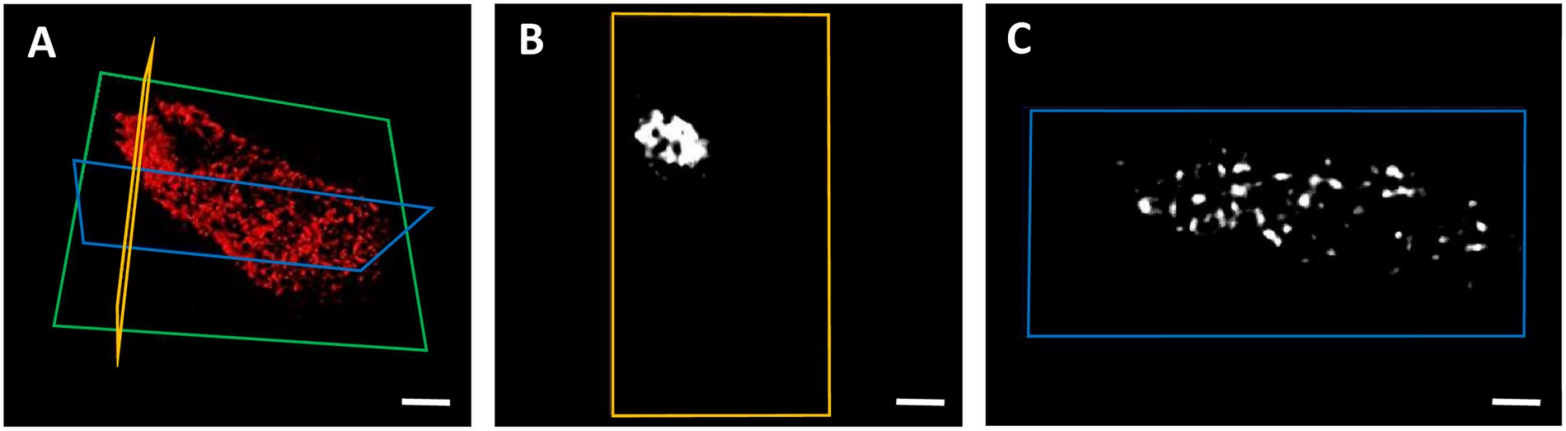
Zinc XRF nanotomography profile of an *E*. *coli* cell. (**A**) Three-dimensional view of the cell. The green box indicates the cell orientation during the tomography data collection. (**B**) Zinc distribution in a slice of the cell through the plane outlined in the yellow box in (**A**) showing the dense distribution of Zn at the polar end of the cell. (**C**) Zinc distribution in a slice of the cell through the plane outlined in blue in (**A**) showing that zinc is unevenly distributed throughout the cell. Scale bar is 500 nm.

The zinc distribution was further investigated in a three-dimensional XRF nanotomography dataset of a single *E*. *coli* cell (Fig. 4). The tomography reconstruction in Fig. 4A as well as the 2D cut in Fig. 4C clearly show that zinc is inhomogeneously distributed throughout the cell. The 2D cut through the cell in Fig. 4B shows that Zn is heavily concentrated at one pole of the cell.

**Figure 5:**
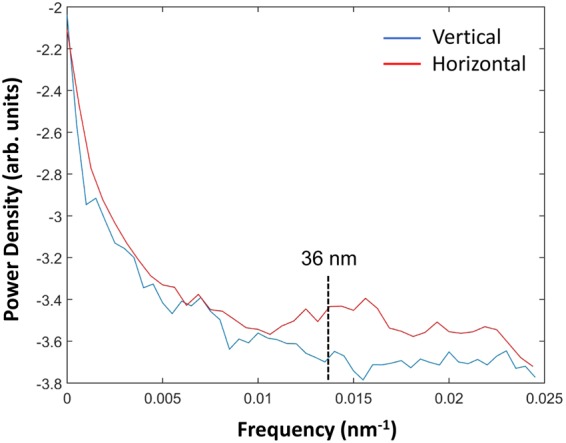
Estimate of the spatial resolution of the Zn XRF *E*. *coli* image using power spectral analysis. The power spectrum signal declines with the spatial frequency before reaching a noise floor. The cutoff point is at a half period of approximately 36 × 36 nm. The Zn XRF image is from a 2D projection using a 20 nm step size and 0.1 s dwell time at 15°.

Since bacteria do not have many well-defined subcellular structures, the spatial resolution of the two-dimensional XRF images was estimated using a power spectral analysis^27,33^. According to Deng *et al*., for low-photon statistics images like XRF images, one can use the fact that Poisson fluctuations (‘shot noises’) are uncorrelated from pixel to pixel so that, at high spatial frequencies, one arrives at a noise floor in Fourier power analysis. Figure 5 shows the result of the power spectral analysis, where we found an estimated spatial resolution of approximately 36 nm (horiz) × 36 nm (vert) using the 2D zinc fluorescence image from the tomography projection at 15*°*.

Another method to estimate the spatial resolution is by using the Fourier Ring Calculation approach which requires two independent datasets acquired under the same conditions^34,35^. Based on previously published work^22^, the beam size routinely produced by the MLLs is about 14 nm x 12 nm when performed with 2D imaging using a well-aligned sample. This results in a depth of focus of ±2 μm for the HXN MLL microscope. However, the rotation stage used for these experiments had a poor run-out error of ~5 μm. Thus, for some rotation angles, the sample moved away from its ideal rotation center. Although the sample was centered in the direction perpendicular to the beam, the run out along the direction parallel to the beam was not corrected. Considering these factors, the XRF image resolution in this study was larger than the published work by Yan, *et al*.^22^.

## Discussion

In this study, X-ray fluorescence microscopy, nanotomography, and ptychography were performed on individual *E*. *coli* cells using a sub-15 nm beam size with a MLL microscope. The distributions of chlorine, calcium, and zinc were imaged, focusing on the sub-cellular distribution of zinc in the cell. *E*. *coli* cells have few internal structures; they possess a nucleoid, which is a highly condensed and membrane-free, irregular structure containing the genetic material of the bacterial cell^36,37^ and a dense distribution of ribosomes. The tomography results showed an inhomogeneous zinc distribution in the cell, with a higher concentration at one pole. Most of the internal labile zinc is thought to be stored in the ribosomes^38–40^. Generally, ribosomes are distributed among and around the central nucleoid. This ribosome distribution leads to ribosome-rich regions especially at the polar end-caps of the cell and in a cylindrical shell surrounding the nucleoid^36^ which would explain the higher amounts of zinc observed in these regions^39–41^.

In rapidly growing *E*. *coli*, it is believed that the majority of the translation events are presumably carried out on mature, freely diffusing mRNAs within this localized ribosome-rich, “protein-factory” region using poly-ribosomes^42^. In bacteria, zinc can play a structural, regulatory, or catalytic role in proteins and is tightly controlled by dedicated systems for high-affinity zinc uptake and export systems involving the cell membranes^40,43^.

One of the challenges of using a MLL microscope is the short working distance between the sample position and the order-sorting aperture (OSA), which was ~0.8 mm in this experiment. Because there is a short working space available to rotate the sample to collect XRF maps at different angles, it was necessary to have a sample and substrate small enough to prevent sample collision with the OSA. To mitigate this, several iterations of sample holders were tested including Mitegen’s Microcrystal Mounts^TM^, direct addition of the cells embedded in NaCl crystals to a tungsten pin, and different sizes of the HXN diving board Si-chip sample holder. In the end, we chose a Si-chip substrate that was less than 0.5 mm wide, which was small enough to safely rotate the sample but large enough to accommodate more than 100 cells for the experiment.

A further limitation of nanoscale X-ray imaging, in particular for living biological specimens, is the inherent radiation damage that occurs with hard X-ray exposure, which can change the structure and chemistry of samples. Generally, samples are measured dried or under cryogenic conditions to mitigate chemical and structural changes when exposed to an intense X-ray beam. With the short working distance of the MLL microscope, sample transfer and data collection at cryogenic conditions presented a significant technical challenge. Instead, we chose to embed the bacteria in small (5–20 µm wide, 1–2 µm thick) sodium chloride crystals, which was a simple and straightforward approach to provide a non-aqueous matrix that retained the three-dimensional structure of individual cells. Furthermore, the XRF and SEM contrast provided by chlorine greatly enhanced the ability to locate individual cells on the sample substrate, also minimizing the radiation dose. While this method may not be applicable to all cells, it is likely to be most effective for many nonadherent cell types including bacteria, archaea, and algae that can easily be suspended in the salt solution prior to drying.

The estimated radiation dose imparted on the sample for the duration of the tomography data collection was 1.3 × 10^9^ Gray. No signs of cell shrinkage or compositional changes were observed. This dose was calculated assuming the main consistency of the cell material was of protein composition of H_48.6_C_32.9_N_8.9_O_8.9_S_0.6_ with a condensed density of 1.35 g/cm^3 ^^44,45^. This dosage was similar to those imparted on biological samples reported by others without seeing any significant mass loss^33,46^, albeit these studies were done under cryogenic conditions.

Having the ability to assess the zinc distribution in *E*. *coli* – as is possible with XRF nanotomography – is essential for establishing the role of zinc in cellular processes and disease. Importantly in bacteria, infectivity can readily be altered by varying levels of zinc in the host system^47^. More broadly, zinc plays a vital role in the physiology of all organisms and its homeostasis is widely studied in areas such as aging, neurodegenerative diseases, cancer, the immune system, and energy metabolism.

This work represents one example of how XRF nanotomography and ptychography can be used to image trace element distribution/concentration and structural morphology, respectively, in biological cells. It was made possible through the development of a synchrotron source with high brightness and coherence, nanofocusing optics, and exceptional beam and sample stability along with a new approach for sample preparation and image collection.

## Conclusions

In summary, this study demonstrates that XRF nanotomography can be used to image the distribution of trace elements in intact biological cells using a sub-15 nm beam in three dimensions, and that simultaneous ptychography can be used for the co-localization of the trace elements with subcellular structures. An approach such as this presents new possibilities in understanding subcellular biochemistry in individual organelles and other subcellular compartments, which are usually analyzed at the organelle population level.
